# Can resisted swimming exercise substitute for the protective effects of estrogen on cardiometabolic risk factors in obese postmenopausal rat model?

**DOI:** 10.22038/ijbms.2025.82005.17745

**Published:** 2025

**Authors:** Sara Shirazpour, Mohammad Khaksari, Abbas Ali Gaini, Hamideh Bashiri, Kayvan Khoramipour, Forouzan Rafie

**Affiliations:** 1 Department of Physiology, Faculty of Medicine, Kerman University of Medical Sciences, Kerman, Iran; 2 Physiology Research Center, Institute of Neuropharmacology, Kerman University of Medical Sciences, Kerman, Iran; 3 Department of Exercise Physiology, University of Tehran, Tehran, Iran; 4 Institute of Neuropharmacology, Neuroscience Research Center, Kerman University of Medical Sciences, Kerman, Iran; 5 i+HeALTH Strategic Research Group, Department of Health Sciences, Miguel de Cervantes European University (UEMC), 47012 Valladolid, Spain; 6 Emory University School of Medicine Division of Geriatrics and Gerontology, Atlanta, Georgia, USA

**Keywords:** 17 beta-estradiol, Bilateral ovariectomies, High-fat diet, Oxidative stress, Swimming

## Abstract

**Objective(s):**

Following our previous studies on the anti-obesity and cardioprotective effects of 17-beta estradiol (E2), this study was designed to determine the effects of Resisted swimming (RSW) training and E2 (alone and in combination) on cardiometabolic risk factors in an obese postmenopausal rat model.

**Materials and Methods:**

Female ovariectomized rats (OVX) were given a standard diet (SD) or a 60% high-fat diet (HFD) for 16 weeks and were divided into two groups: SD and HFD. The rats were divided into ten groups to assess the effects of 8 weeks of E2 (1 mg/kg, IP) administration and RSW (5 days a week) on cardiometabolic risk factors. Parameters including body weight, BMI, visceral fat, blood glucose (BG), and cardiac oxidative stress were assessed 72 hr after the last swimming session.

**Results:**

HFD increased body weight, BMI, visceral fat, and BG levels in OVX rats. Additionally, it negatively affected the lipid profile and cardiac oxidative stress, but both E2 and RSW reduced these parameters in HFD-fed OVX rats. Although RSW and E2 equally prevented these changes, swimming was more effective than estrogen in increasing HDL levels in the SD group. The combination of E2 and RSW had a more significant effect on modulating glucose, TAC, TG, and HDL indices than the individual treatments.

**Conclusion:**

Overall, RSW ameliorates cardiometabolic risk factors in postmenopausal conditions caused by obesity, probably by modulating cardiac oxidative stress. It is also an effective non-pharmacological treatment for E2 substitution.

## Introduction

Currently, about one-third of the global population is affected by obesity and overweight (1). Obesity, defined by a body mass index (BMI) ≥ 30 kg/m², is a chronic condition that is increasing at an alarming rate, particularly among women (2). It is projected that by 2025, the prevalence of obesity will reach 18% in men and over 21% in women (2). With the increasing prevalence of obesity, its associated cardio-metabolic disorders, such as diabetes, hypertension, and dyslipidemia, are also on the rise worldwide (3). The principal factors contributing to obesity are reduced physical activity and consumption of a high-fat diet (HFD), both of which lead to fat accumulation (4, 5). This accumulation elevates the lipid profile (6), generates reactive oxygen species (ROS), and diminishes total antioxidants (7). In addition, the cardiac redox imbalance is associated with increased lipid profile and blood glucose (BG) levels (8).

Postmenopausal women are more susceptible to weight gain, dyslipidemia, and hyperglycemia compared to their premenopausal counterparts (9). They also experience a four-fold incidence of obesity-related cardiovascular disorders (CVD) (10). During menopause, the most significant hormonal changes involve 17-beta estradiol (E2), an ovarian hormone crucial in regulating blood pressure, lipid metabolism, metabolic homeostasis, and cardiac function (7). Postmenopausal estrogen deficiency is associated with reduced energy expenditure, which leads to increased visceral fat mass and metabolic disorders (11). This deficiency also alters triglyceride (TG) levels, LDL levels, and oxidative stress parameters associated with CVD in postmenopausal women (12).

As obesity is a significant risk factor in cardiometabolic diseases, weight loss through regular exercise is an effective non-pharmacological treatment (13). Regarding weight loss in obese individuals with CVD, aquatic exercises are preferred over other forms of exercise (14). Despite people’s high body weight in aquatic exercises, water’s reduced density and buoyancy lead to higher calorie expenditure and reduced fatigue levels than land-based exercises (14, 15). Aquatic exercises like swimming can more effectively impact body composition and weight loss by enhancing muscle strength and promoting fat-burning and cardiovascular endurance (15). RSW, which involves the addition of loads during swimming, engages a greater number of muscle groups and promotes increased muscle mass growth (16). Therefore, it has a greater effect on reducing BG levels and lipid profile (17). It has been demonstrated that swimming reduces serum levels of TG, TC, LDL, and glucose in obese rats with metabolic syndrome (18). It also increases HDL (19) and reduces cardiac oxidative stress (20) in young obese female rats. In addition, aquatic exercises during post-menopause reduce lipid profile, cardiovascular disease CVD, and oxidative stress through mechanism(s) such as promoting enzymatic activity related to fat breakdown (21), reductions in arterial stiffness (22), maximal aerobic capacity (23), strengthening the cardiac muscle (22), and increasing the activity of superoxide dismutase (SOD) and glutathione peroxidase (24).

In our previous study, we found that estrogen reduces cardiometabolic disorders (5), and also swimming has been reported to play a role in preventing CVD. Although the cardiometabolic effects of swimming and E2 have been reported individually, the combined effects of E2 and RSW training on obesity and cardio-metabolic risk factors in postmenopausal conditions have not been reported. Therefore, we designed the present study to examine the effects of RSW and E2 (alone and in combination) on the body weight, BMI, visceral fat, BG levels, and lipid profile of ovariectomized obese rats. This study also aimed to measure the effects of these treatments on changes in cardiac oxidative stress parameters in these animals to evaluate the related mechanisms.

## Materials and Methods

### Animals

We obtained adult female Wistar rats aged 3–4 months and weighing 200–250 g from animal care and breeding of the Kerman University of Medical Sciences Animal Center in Kerman, Iran. The rats were housed in a controlled environment, maintaining a temperature of 23–25 °C, a 12-hour light-dark cycle, and free access to food and water. All experimental protocols adhered to the National Institute of Health guidelines for the Care and Use of Laboratory Animals and were approved by the Institutional Animal Care and Use Committee of Kerman University (No. IR.KMU.AEC.1402.052).

### Experimental protocol

**Figure 1:**
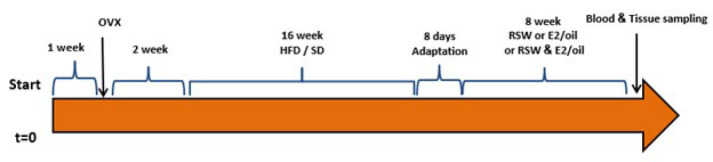
A Graphic depicting the experimental protocol, which include ovariectomy, obesity induction, and treatment with RSW and E2 in ovariectomized rats

Female ovariectomized rats (OVX) were divided into two main groups, SD and HFD, and were given either a standard diet (SD) or a 60% high-fat diet (HFD) for 16 weeks. To assess the effects of 8 weeks of E2 injection and swimming on cardiometabolic risk factors, the OVX rats were divided into ten groups (n=6/group) at the end of 16 weeks: 1) SD+Cont, 2) HFD+Cont, 3) SD+Oil (E2 solvent (1 mg/kg), 4) HFD+Oil (1 mg/kg E2 solvent), 5) SD+E2 (1 mg/kg E2), 6) HFD+E2 (1 ml/day E2), 7) SD+ RSW+Oil (1 mg/kg E2 solvent and resisted swimming), 8) HFD+RSW+Oil (1 mg/kg E2 solvent and resisted swimming), 9) SD+RSW+E2 (1 mg/kg E2 and resisted swimming), and 10) HFD+RSW+E2 (1 mg/kg E2 and resisted swimming). E2 and sesame oil (1 mg/kg) were injected in rats intraperitoneally (IP) every four days (to mimic the natural estrous cycle) for 8 weeks (25, 26), and resisted swimming was done 5 days a week for 8 weeks (Figure 1).

### Drug

Ketamine and xylazine were acquired from Vetased Company (Utrecht, the Netherlands), and 17-β estradiol (E2) and sesame oil were obtained from Aburaihan Pharmaceutical Company (Tehran, Iran).

### Bilateral ovariectomy

The animals were anesthetized using an intraperitoneal dose of 80/10 mg/kg of ketamine and xylazine. Then, a small longitudinal incision was made in the abdomen. The ovaries were exposed and removed after opening the skin, abdominal muscles, and fascia. Finally, the skin and muscles were sutured, and 2 ml of saline solution was injected into the peritoneum abdominal. Two weeks following OVX, the following tests were conducted (27).

### Dietary obesity induction

Animals were fed a high-fat diet (HFD) for 16 weeks, as outlined in Table 1 (5, 28). The high-fat diet was sourced from the Royan Institute in Isfahan, Iran (5). This table shows the percentages of ingredients in standard and high-fat diets. The HFD has higher fat and lower carbohydrate percentages than a standard diet, but the other components are the same.

### Analysis of body mass index (BMI) and changes in body weight

The body weight of the animals was assessed weekly. The body mass index (BMI) and body weight changes (%) were calculated every two months using the following equations(5):

BMI = body weight (g) / length (cm^2^)

Body weight changes (%) = (Final body weight - Initial body weight / initial body weight) × 100

### Swimming protocol

Following the induction of obesity, all rats in the swimming groups underwent an eight-day water and swimming adaptation (29) (detailed in Table 2) in a swimming pool 100 cm in length, 80 cm in width, and 80 cm in height, filled with 50 cm of warm water (30 °C). The lactate threshold was determined using the protocol from Gobatto *et al*. (30), establishing that a load of 1% of a rat’s body weight corresponds to the lactate threshold. Therefore, the intensity in this study was classified as low (between 0% and 1% of body weight). Subsequently, the primary training program, outlined in Table 3, consisted of 8 weeks of swimming conducted in 5 weekly sessions, each lasting 30 to 45 min, with a load of 0–1% of the rats’ body weight. During the training period, the principle of overload was implemented by progressively increasing the duration of the sessions. After the training sessions, the rats were dried and returned to standard conditions (31). 

### Collections of visceral adipose tissue

Seventy-two hours after the last swimming session, the animals were sacrificed under deep and irreversible anesthesia and all visceral adipose depots around internal organs were removed, and relative white adiposity was determined using the formula (sum of fat pad weights) / (body weight) x 100 (11)

### Assessment of lipid profile and blood glucose levels

Seventy-two hours after the last swimming session, Blood samples were obtained for biochemical evaluation, and serum was separated from the blood samples by centrifuging at 4 °C and 3000 rpm for 10 min. Blood glucose (BG; mg/dL) was measured monthly. At the end of the experiment, the lipid profile, encompassing triglycerides (TG), total cholesterol (TC), high-density lipoprotein (HDL), and low-density lipoprotein (LDL) (mg/dL), was analyzed using an autoanalyzer (MINDRAY, China) and commercial biochemical kits from Pars Azmoon (Iran) (5).

### Assessment of oxidative stress parameters

Seventy-two hours after the last swimming session, total anti-oxidant capacity (TAC) and malondialdehyde (MDA) were calculated using an automated microplate reader (BioTek, USA) in conjunction with commercial biochemical assay kits (ZellBio, Germany). TAC has a sensitivity of 0.5 U/ml and an assay range of 1–100 U/ml, and MDA has a sensitivity of 0.1 µM and an assay range of 0.78–50 µM. Oxidative stress parameters were measured in the cardiac muscle extract (5, 32).

### Statistical analysis

All statistical analyses were conducted using GraphPad Prism 6.0 (GraphPad Software, San Diego, CA, USA). Data normality was assessed with the Shapiro-Wilk W test. Differences in body weight changes, BMI, BG levels, and lipid profiles between the SD and HFD groups were evaluated using t-tests. Two-way ANOVA followed by Tukey’s post hoc test was employed to compare body weight, BMI, BG, visceral fat, lipid profiles, and oxidative stress parameters across groups. Results are presented as mean ± SEM, with *P*<0.05 considered statistically significant.

## Results

### The effects of different diets on body weight in OVX animals

**Figure 2:**
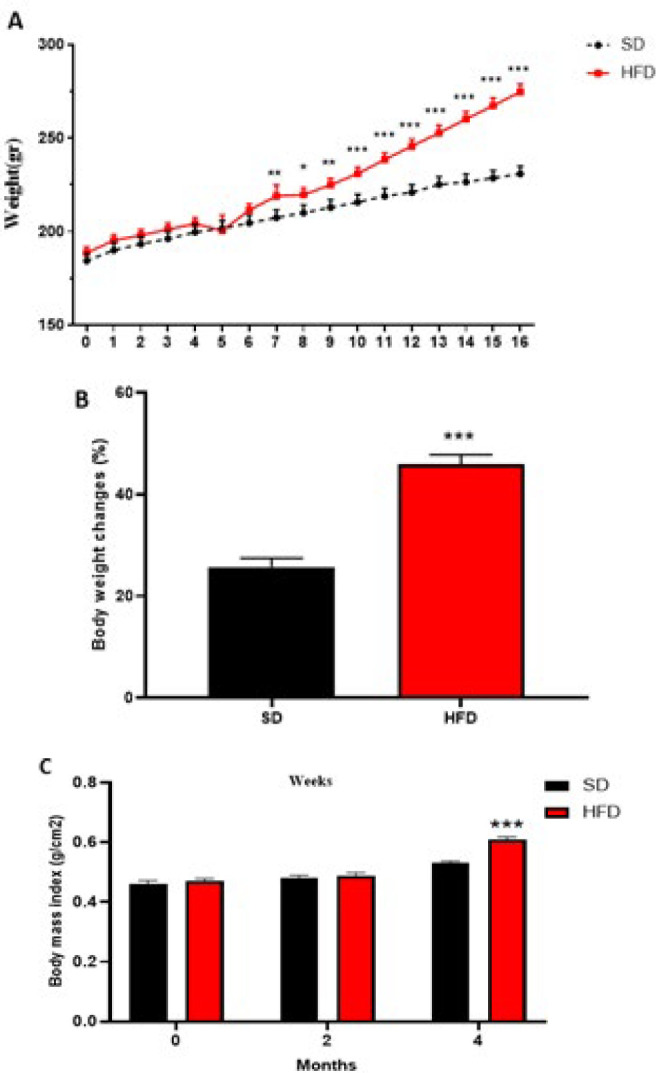
Effects of different diets on (A) body weight, (B) body weight changes and (C) BMI in the SD and HFD groups of ovariectomized rats (n = 6)

The body weight and BMI of OVX animals are shown in Figures 2A–C. No significant weight gain differences were observed between the SD and HFD groups until week 7. A high-fat diet leads to weight gain, so body weight from week 7 to week 16 significantly increased in the HFD group compared to the SD group (*P*<0.01 and *P*<0.001) (Figure 2A). Body weight changes in the HFD rats markedly increased as compared to the SD rats in the 16th week after the induction of dietary obesity (*P*<0.001) (Figure 2B). Also, a markedly elevated BMI was observed in the HFD group in comparison with the SD group in month 4 after the induction of dietary obesity (*P*<0.001) (Figure 2C). 

### Effects of RSW and E2 (alone and in combination) on body weight in animals fed different diets

**Figure 3:**
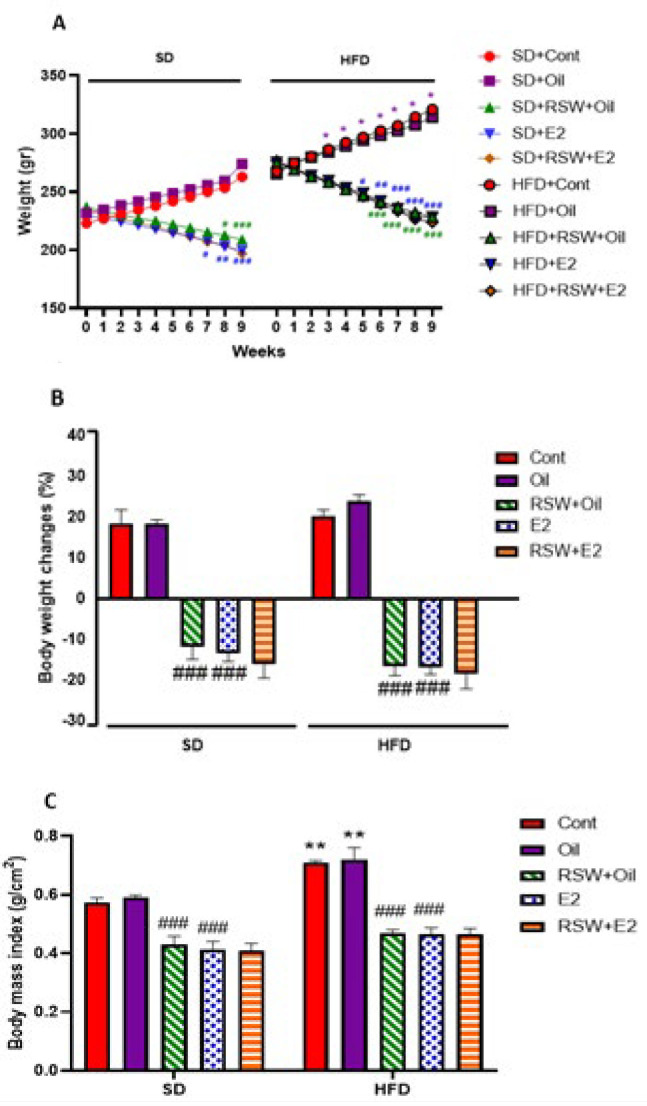
Effects of RSW and E2 therapy alone or in combination on (A) body weight, (B) body weight changes and (C) BMI in different experimental groups of ovariectomized rats (n = 6)

Figure 3A indicates that the body weight from week 4 to week 8 was significantly increased in HFD+Oil groups compared to the SD+Oil group (*P*<0.05). A decrease was observed in body weight in the RSW +SD group in comparison with the SD+Oil group at 8 weeks (*P*<0.05) and 9 weeks (*P*<0.001) treatment with swimming. Also, the body weight of RSW-treated rats that received HFD from week 6 to week 9 was reduced in comparison with the HFD + Oil group (*P*<0.01 and *P*<0.001 ), and the body weight in the both SD + E2 and HFD + E2 groups was less than the same oil-treated groups in week 5 (*P*<0.05), week 6 (*P*<0.001), week 7 (*P*<0.05, *P*<0.001; respectively), week 8 (*P*<0.01, *P*<0.001; respectively) and week 9 (*P*<0.001) treatment with E2.

Figure 3B illustrates that treatment with RSW+Oil caused weight loss in both SD (-11.85) and HFD (-16.68) groups in comparison with the same oil groups (*P*<0.001), and reductions in body weight changes were observed in SD and HFD treated with E2 compared to the same oil groups (*P*<0.001). The BMI in the HFD+Cont and HFD+Oil groups considerably increased compared to the SD+Cont (*P*<0.01) and the SD+Oil (*P*<0.01) groups, and treatment with RSW or E2 in both SD and HFD groups significantly decreased the BMI in comparison with the same oil groups (*P*<0.001) (Figure 3C).

### Effects of RSW and E2 (alone and in combination) on visceral adiposity

**Figure 4:**
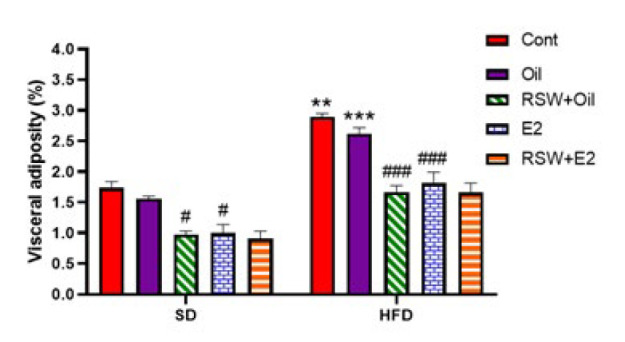
Effects of RSW and E2 therapy alone or in combination on visceral adiposity (%) in different experimental groups of ovariectomized rats (n = 6)

The results in Figure 4 show that the percentage of visceral adiposity in the HFD+Cont and HFD+Oil groups increased considerably compared to the SD+Cont (*P*<0.01) and SD+Oil (*P*<0.001) groups, and there was no significant difference in the visceral adiposity (%) between the SD+Oil and HFD+Oil groups with the same Cont groups. In addition, the visceral adiposity (%) was significantly lower in the RSW+SD group and RSW+HFD group as compared with the same oil groups (*P*<0.05 and *P*<0.001, respectively). Also, the percentage of visceral adiposity was reduced in SD and HFD treated with E2 compared to the same oil groups (*P*<0.001 and *P*<0.001, respectively). 

### Effect of different diets on BG in OVX animals

**Figure 5:**
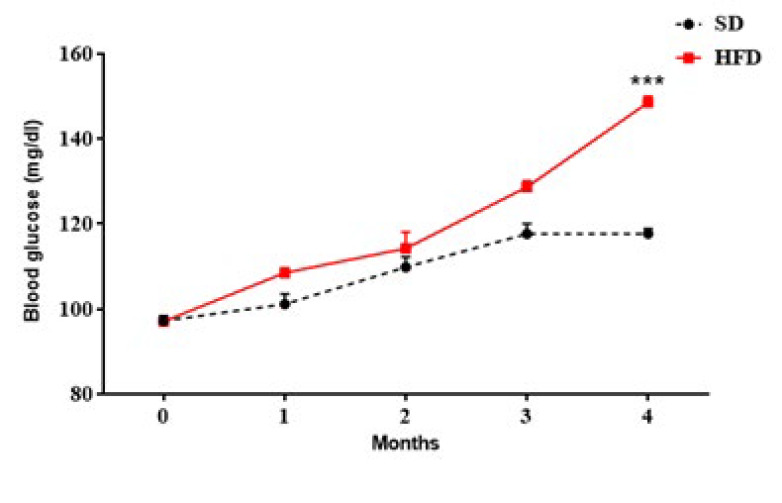
Effects of different diets on BG level (mg/dl) in the SD and HFD groups of ovariectomized rats (n = 6)

As shown in Figure 5, the consumption of HFD resulted in elevated serum BG levels from the 16th week compared with the utilization of SD in OVX rats (*P*<0.001).

### Effects of RSW and E2 (alone and in combination) on BG in animals fed different diets

**Figure 6:**
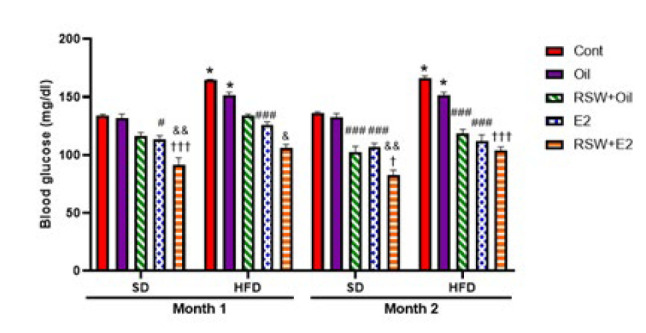
Effects of RSW and E2 therapy alone or in combination on BG level (mg/dl) in different experimental groups of ovariectomized rats (n = 6)

 The amount of BG changes in experimental groups at different times after induction of obesity is shown in Figure 6. The level of BG significantly increased in the HFD+Cont and HFD+Oil groups compared to the SD+ Cont (*P*<0.05) and SD+Oil (*P*<0.05) groups at both times. However, there was no significant difference in the amount of BG between the SD+Oil and HFD+Oil groups and the same Cont groups. The reduction of the BG level was observed in SD and HFD rats treated with RSW+Oil compared to the same oil groups (*P*<0.001) in the eighth week. A considerable decrease was observed in BG levels in the E2+SD and E2+HFD groups in comparison with the same oil groups after four weeks (*P*<0.05 and *P*<0.001, respectively) and eight weeks (*P*<0.001 and *P*<0.001; respectively) after induction of obesity. Treatment with RSW+E2 in the SD and HFD groups significantly decreased the level of BG in comparison with the same RSW+Oil groups in the first month (*P*<0.001) and in the second month (*P*<0.05). Furthermore, in the RSW+E2 group, BG levels were significantly decreased compared with the E2+SD and E2+HFD groups after 4 weeks (*P*<0.01) and after 8 weeks (*P*<0.05) after induction of obesity.

### Effect of different diets on lipid profile in OVX animals

**Figure 7:**
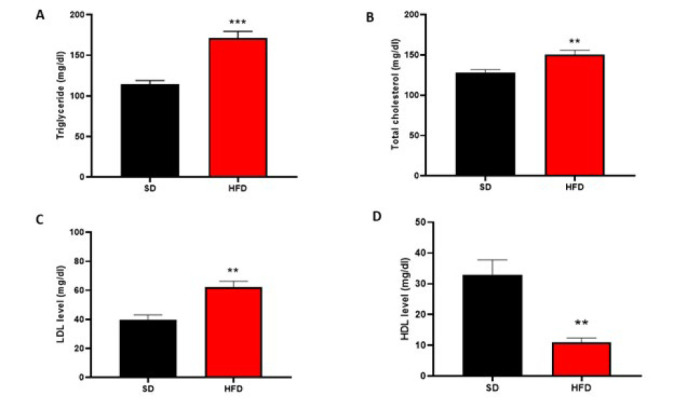
Effects of different diets on serum levels (mg/dl) of: (A)TG, (B)TC, (C) LDL and (D) HDL in the SD and HFD groups of ovariectomized rats (n = 6)

The results of rats’ lipid profiles in OVX animals are shown in Figure 7 (A-D). HFD elevated serum levels of TG, TC, and LDL and reduced serum levels of HDL in comparison with SD in OVX rats (*P*<0.001, *P*<0.01, *P*<0.01, and *P*<0.01, respectively).

### Effects of RSW and E2 (alone and in combination) on lipid profile in animals fed different diets

**Figure 8:**
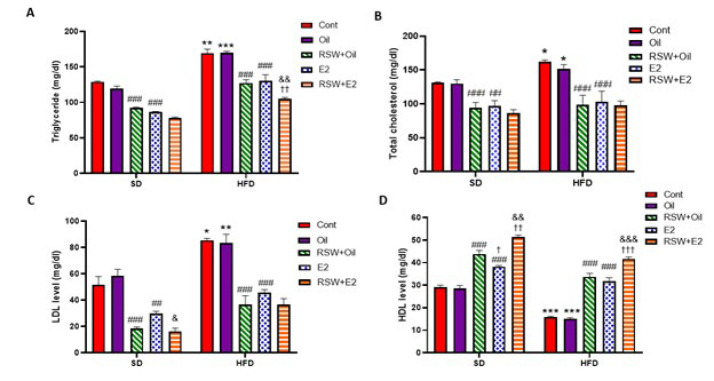
Effects of RSW and E2 therapy alone or in combination on serum levels (mg/dl) of: (A) TG, (B) TC, (C) LDL, and (D) HDL in different groups of ovariectomized rats (n = 6)

Figure 8A-D results show that TG, TC, and LDL serum levels significantly increased. The serum level of HDL considerably decreased in the HFD+Cont and HFD+Oil groups compared to the SD+ Cont (*P*<0.01, *P*<0.05, *P*<0.05, and *P*<0.001, respectively) and SD+Oil groups (*P*<0.001, *P*<0.05, *P*<0.01, and *P*<0.001, respectively). There was no significant difference in serum lipid profile between the SD+Oil and HFD+Oil groups and the same Cont groups. As shown in Figure 8A, serum TG levels were reduced in both SD and HFD groups treated with RSW or E2 compared to the same oil groups (*P*<0.001). Also, the serum level of TG decreased in the SD+RSW+E2 group compared with the SD+RSW+Oil (*P*<0.001) and the SD+E2 (*P*<0.01) groups. Treatment with RSW+Oil or E2 considerably decreased the serum level of TC in the SD and HFD groups compared with the same oil groups (*P*<0.001). However, there was no significant difference in the serum TC level between the RSW+E2 (SD/HFD) group and the RSW+Oil (SD/HFD) or E2 (SD/HFD) groups (Figure 8B). The serum LDL level was changes similar to the serum TC level changes, so both treatments were effective (*P*<0.001). However, treatment with RSW+E2 considerably decreased the LDL serum level in the SD group compared with the E2 groups (*P*<0.05) (Figure 8C). Treatment with RSW+Oil or E2 significantly increased HDL serum levels in both SD and HFD groups compared to the same oil groups (*P*<0.001). In addition, a marked increase in serum HDL level was observed in the SD+RSW+E2 group compared to the SD+RSW+Oil (*P*<0.01) and SD+E2 (*P*<0.001) groups. Also, treatment with RSW+E2 in the HFD group considerably increased the serum level of HDL in comparison with the RSW+Oil (*P*<0.001) and E2 (*P*<0.001) groups (Figure 8D).

### Effect of RSW and E2 (alone and in combination) on cardiac oxidative stress parameters in animals fed different diets

**Figure 9:**
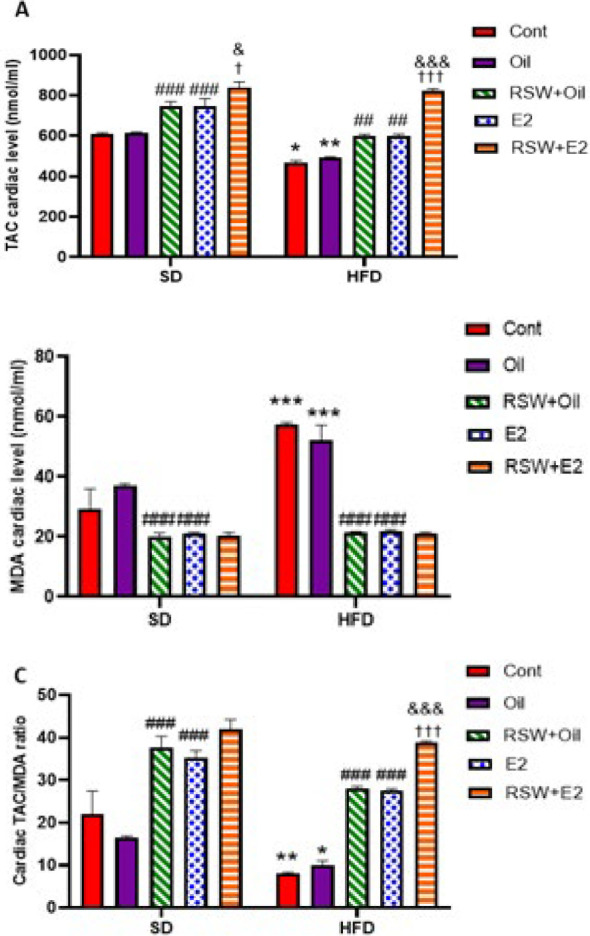
Effects of RSW and E2 therapy alone or in combination on cardiac levels of: (A) TAC (nmol/ml), (B) MDA (nmol/ml), and (C) TAC/MDA ratio in different groups of ovariectomized rats (n = 6)

As shown in Figure 9A-C, a marked decrease was observed in cardiac TAC activity, and the cardiac TAC/MDA ratio, and a significantly increased cardiac MDA level was observed in the HFD+Cont group compared to the SD+Cont group (*P*<0.05, *P*<0.01, and *P*<0.001, respectively). Cardiac TAC level was significantly decreased, and cardiac MDA level was significantly increased in the HFD+Oil group compared to the SD+Oil group (*P*<0.01 and *P*<0.001, respectively). However, no significant difference was shown in the amount of these oxidative stress parameters between the HFD+Oil and SD+Oil groups and the same Cont groups. Figure 9A indicates that treatment with RSW+Oil or E2 considerably elevated the cardiac TAC activity in both SD and HFD groups compared with the same oil groups (*P*<0.001 and *P*<0.01, respectively). Also, treatment with RSW+E2 in the SD group significantly increased the cardiac level of TAC in comparison with RSW+Oil (*P*<0.05) and E2 (*P*<0.05) groups. A markedly increased cardiac TAC activity was observed in the HFD+RSW+E2 group compared to the HFD+RSW+Oil (*P*<0.001) and HFD + E2 (*P*<0.001) groups. A reduction in the cardiac level of MDA was observed in SD and HFD treated with RSW+Oil or E2 compared to the same oil groups (*P*<0.001). However, there was no significant difference in the cardiac MDA level between the RSW+ 2 (SD/HFD) group and the RSW+Oil (SD/HFD) or E2 (SD/HFD) groups (Figure 9B). The elevation of the cardiac TAC/MDA ratio was observed in both SD and HFD groups treated with RSW or E2 compared to the same oil groups (*P*<0.001). Also, the cardiac TAC/MDA ratio increased in the HFD+RSW+E2 group compared with the SD+RSW+Oil and SD+E2 groups (*P*<0.001) (Figure 9C).

### Effects of RSW on body weight, blood glucose, lipid profile, and oxidative stress parameters as compared to E2

The results in Table 1 showed that the serum levels of HDL significantly increased in the RSW+SD group compared to the E2+SD group (*P*<0.05). There were no significant differences between the RSW (SD/HFD) and E2 (SD/HFD) groups in TG, TC, LDL, body weight, visceral adiposity, blood glucose, and oxidative stress. This means that RSW could be a substitute with E2 in OVX rats.

## Discussion

Resistant swimming delays the progression of CVD risk factors. In this study, the protective effects of RSW alone and in combination with estrogen therapy on obesity-related cardiometabolic risk factors have been investigated in OVX rats (postmenopausal mode). In all variables studied in this research, RSW can be considered a good substitute for E2 in OVX rats, and a combination of estrogen and RSW can enhance the effects of each intervention alone on modulating glucose, TAC, TG, and HDL.

OVX: Ovariectomy, HFD: High-fat diet, SD: Standard diet, RSW: Resisted swimming, E2: 17-β Estradiol, Oil: Sesame oil

**Table 1:** Ingredients of the standard diet (SD) and high-fat diet (HFD) for experimental groups in ovariectomized rats

Ingredients	Standard diet	High-fat diet
Fat	10%	60%
Carbohydrate	70%	20%
Protein	19%	19%
Fiber, Mineral, Vitamins	1%	1%
Total energy	341 Cal/100	429 Cal/100

**Tabel 2:** The stages of adaptation to water and swimmingin in the training groups

Swimming adaptation	Water adaptation	Days
	5 min in a swimming pool with water reaching the rats' feet	1
	5 min in a swimming pool with water reaching the rats' heads	2
	5 min in a swimming pool with water passing over the rats' heads occasionally as they swim	3
5 min swimming in the pool		4
10 min swimming in the pool		5
15 min swimming in the pool		6
20 min swimming in the pool		7
25 min swimming in the pool		8

**Table 3:** Swimming protocol in the training groups

Workload (% of body weight)	Duration (min)	Weeks
0	30	1
1	30	2
1	35	3
1	35	4
1	40	5
1	40	6
1 1	45 45	7 8

**Table 4:** Effect of swimming on body weight, visceral adiposity, blood glucose, lipid profile, and oxidative stress parameter compared to E2 in ovariectomized rats

** Groups**	**BW** **(gr)**	**VA** **(%)**	**BG** **(mg/dl)**	**TG (mg/dl)**	**TC** **(mg/dl)**	**LDL (mg/dl)**	**HDL (mg/dl)**	**TAC (nm/ml)**	**MDA (nm/ml)**
**SD + RSW**	208.8±10.7	0.97±0.05	102.6±4.8	92.5±0.74	94.2±3.5	18.5±0.84	43.86^*^±1.5	745.8±23.8	20±1.1
SD + E2	199.4±14.6	0.99±0.14	106.6±3.3	86.7±0.37	96.6±3.7	29.8±1.7	38.24±0.48	748.9±78.9	21.16±0.34
***P*** **-value**	*P*=0.997	*P*>0.999	*P*>0.999	*P*=0.984	*P*>0.999	*P*>0.768	*P*=0.036	*P*>0.999	*P*>0.999
**HFD+ RSW**	223.8±7.8	1.66±0.10	118.5±3.4	127.4±4.6	98.6±6.2	36.5±6.7	33.7±1.5	600.3±16	21.34±0.35
**HFD + E2**	228.4±5.8	1.81±0.17	112±5.2	130.4±8.6	103±6.9	33.7±2.3	31.8±1.5	598.5±24.4	21.74±0.54
***P*** **-value**	*P*>0.999	*P*=0.995	*P*>0.998	*P*>0.999	*P*=0.998	*P*>0.894	*P*=0.969	*P*>0.999	*P*>0.999

Data are presented as mean ± SEM. n=6. *P<0.05 VS E2+SD. SWIM: Swimming, E2: 17-β estradiol, HFD: High-fat diet, SD: Standard diet, BW: Body weight, VA: Visceral adiposity

The data are presented as mean ± SEM. ***P<0.001 and **P<0.01 VS SD. HFD: High-fat diet, SD: Standard diet, BMI: Body mass index

The data are presented as mean ± SEM. **P<0.01 and *P<0.05 VS Cont +SD or Oil+SD; ###*P*<0.001, ##*P*<0.01 and #*P*<0.05 VS The same oil groups. RSW: Resisted swimming, E2: 17-β estradiol, HFD: High-fat diet, SD: Standard diet, BMI: Body mass index.

The data are presented as mean ± SEM. ***P<0.001 and **P<0.01 VS Cont +SD or Oil+SD; ###*P*<0.001 and #*P*<0.05 VS the same oil groups. RSW: Resisted swimming, E2: 17-β estradiol, HFD: High-fat diet, SD: Standard diet.

, BG: Blood glucose, TG: Triglyceride, TC: Total cholesterol, LDL: Low-density lipoprotein, HDL: High-density lipoprotein, TAC: Total antioxidant capacity, MDA: Malondialdehyde.

The data are presented as mean ± SEM. ***P<0.001 VS SD. HFD: High-fat diet, SD: Standard diet, BG: Blood glucose.

The data are presented as mean ± SEM. *P<0.05 VS Cont+SD or Oil+SD; ###*P*<0.001, ##*P*<0.01, and #*P*<0.05 VS the same oil groups; †††*P*<0.001 and †*P*<0.05 VS the same RSW +Oil groups; &&*P*<0.01 and &*P*<0.05, VS the same E2 groups. RSW: Resisted swimming, E2: 17-β estradiol, HFD: High-fat diet, SD: Standard diet, BG: Blood glucose.

The data are presented as mean ± SEM. ***P<0.001 and **P<0.01 VS SD. HFD: High-fat diet, SD: Standard diet, TG: Triglyceride, TC: Total cholesterol, LDL: Low-density lipoprotein, HDL: High-density lipoprotein.

The data are presented as mean ± SEM. **P<0.01 and *P<0.05 VS same SD or Oil groups; ###*P*<0.001, ##*P*<0.01, and #*P*<0.05 VS same Oil groups; ††† *P*<0.001, †† *P*<0.01 VS same RSW +Oil groups. &&& *P*<0.001, && *P*< 0. 01 VS same E2 groups. RSW: Resisted swimming, E2: 17-β estradiol, HFD: High-fat diet, SD: Standard diet, TG: Triglyceride, TC: Total cholesterol, LDL: Low-density lipoprotein, HDL: High-density lipoprotein.

The data are presented as mean ± SEM. ***P<0.001, **P<0.01, and *P<0.05 VS Cont+SD or Oil+SD; ###*P*<0.001 and ##*P*<0.01 VS the same oil groups. †††*P*<0.001 and †*P*<0.05 VS The same RSW +Oil groups; &&&*P*<0.001 and &*P*<0.05, VS The same E2 groups. RSW: Resisted swimming, E2: 17-β estradiol, HFD: High-fat diet, SD: Standard diet, TAC: Total antioxidant capacity, MDA: Malondialdehyde.

### Body weight

The Results of this study indicate that in addition to ovariectomy, which leads to weight gain in both SD and HFD groups, a high-fat diet also increases body weight, BMI, and visceral fat in OVX rats. Both RSW and estrogen therapy reduced the indices elevated by ovariectomy and a high-fat diet. Although there was no difference between E2 therapy and RSW, swimming can be considered a substitute for estrogen in an obese postmenopausal rat model. On the other hand, our results showed that RSW did not enhance the effects of E2 on reducing body weight, BMI, and visceral fat, indicating no additive effect between these two treatments.

Consistent with our research, It has been documented that ovariectomy leads to fat accumulation, particularly visceral fat, likely due to a decreased basal metabolism and an increased susceptibility to fat accumulation (33). In addition, consistent with our study, it has been shown that rats consuming a high-fat diet are more susceptible to obesity (5). The increased obesity due to HFD may be attributed to reduced energy expenditure through central mechanisms (33). In line with our findings, it has also been documented that swimming reduces obesity induced by HFD and ovariectomy (34). Studies show that compared to other exercises, swimming reduces body weight, visceral fat, and BMI more effectively in postmenopausal women by activating PPARα in the liver and skeletal muscle (34, 35). 

Also, in line with our study, it has been demonstrated that E2 reduces body weight, BMI, and abdominal fat in obese ovariectomized rats who consumed an HFD for 16 weeks, as reported by Hajializadeh *et al*. (5). Some of the potential mechanisms of E2 in body weight loss include reducing metabolism (energy expenditure) (36), inflammation (11), and orexigenic signaling (37), enhancing the appetite-suppressing effects of leptin and cholecystokinin (37). 

In agreement with our study, Claudio *et al*. showed that 8 weeks of swimming and E2 therapy effectively reduced body weight in ovariectomized rats (38). However, in contrast to our findings, the combined effect of exercise and E2 on increased weight loss compared to each one alone has been reported in ovariectomized rats (39). Possible reasons for these differences could include the type of exercise used (endurance or resistance), the sex of the animal, the species of the animal, and the length of the drug treatment (39).

### Blood glucose

The findings of this investigation demonstrated that after one month of therapeutic interventions, estrogen alone reduced hyperglycemia induced by ovariectomy and obesity in both SD and HFD diets. However, since this effect in the combined RSW and E2 groups was more significant than in the E2 group, it can be concluded that exercise enhanced the effects of estrogen after one month in both the SD and HFD groups. Conversely, at the end of the two-month study, RSW and estrogen effectively reduced hyperglycemia induced by the SD and HFD diets, indicating that exercise could substitute estrogen. In the combined group, the effect of E2 was more significant only in the SD group.

In line with our study, it has been shown that the increase in fasting glycemia levels in ovariectomized rats consuming a high-fat diet can be linked to elevated insulin resistance and decreased insulin sensitivity caused by excessive deposition of visceral fat (40, 41). Habibi *et al*. showed in a study that dysfunction of insulin secretion caused by estrogen reduction improved after 8 weeks of swimming (42). Additionally, it has been reported that swimming is an effective exercise strategy for enhancing insulin sensitivity and glucose control in postmenopausal women (43). Swimming has a more significant effect on reducing BG levels by increasing muscle mass (35). Some evidence suggests that swimming can improve hyperglycemia by reducing inflammation in the pancreatic beta cells (42) and increasing the number and activity of GLUT-4 in skeletal muscle (44).

Furthermore, in line with our findings, evidence shows that E2 treatment lowers BG levels in ovariectomized rats fed an HFD (5) and positively affects insulin regulation (26). Additionally, it has been reported that aerobic exercise combined with E2 can correct insulin resistance in skeletal muscle glucose transport in OVX animals (40). Contrary to our study, it has been shown that aerobic exercise does not modulate insulin function in estrogen-treated OVX animals. The possible reasons for these conflicting results can be differences in the type of exercise and the dosage of estrogen used (40).

### Lipid profile

Examination of the lipid profile in this study showed that HFD caused a more significant increase in TC, TG, and LDL serum levels and decreased serum HDL levels compared to SD. At the same time, both RSW and estrogen prevented these changes in both diets. Although there was no significant difference between the effects of RSW and E2 on modulating TC, TG, and LDL serum levels, RSW had a more significant effect than estrogen on increasing HDL serum levels in the SD group. Therefore, RSW has the potential to be a better substitute for estrogen in ameliorating defects in the lipid profile associated with obesity in a postmenopausal rat model. Simultaneous use of estrogen and RSW intensified the effects of each intervention alone, particularly on moderating TG and HDL.

Consistent with our study, consumption of HFD and induction of ovariectomy in rats have been shown to elevate TC, TG, and LDL levels and reduce HDL serum levels (5). Excessive deposition of visceral fat caused by ovariectomy and HFD leads to lipid profile defects (45), which swimming was able to improve in this study (46). Furthermore, a study documented that regular swimming effectively improves blood lipid levels in postmenopausal women (21). It has also been shown that resistance aerobic exercise significantly reduces the serum levels of TC, TG, and LDL and increases the serum level of HDL (17). This occurs because, along with the prolonged activation of large muscles, the increase in muscle mass also enhances the capacity of skeletal muscle to utilize lipids (17, 47). Other possible mechanisms for the modulation of lipid profile by swimming include up-regulation of genes related to fatty acid transport (48), activation of PPARα in the liver (34), and regulation of lipid metabolism through the PANDER-AKT pathway (48).

In line with our findings, it has been reported that treatment with E2 improves lipid profile disturbances (5), consequently reducing TC, TG, and LDL serum levels, increasing HDL serum levels, and reversing dyslipidemia induced by ovariectomy (5, 12). E2 also improves the lipid profile by reducing body weight (49), fat deposition (49), and hepatic insulin resistance (40). 

It has been reported that the combination of estrogen and aerobic exercise effectively reduces HDL oxidation in postmenopausal women (50). In addition, regular aerobic exercise combined with an E2 injection has been shown to correct the lipid profile deficiency in OVX animals (40). In contrast to our findings, it has been reported that treadmill training cannot enhance the effects of estrogen on increasing HDL (51). This may be due to differences in E2 dosage and the type of exercise, as the exercise regimen used in the present study is a type of resistance-based exercise. At the same time, the treadmill is an endurance-based exercise (51).

### Oxidative stress

Regarding changes in oxidative stress, we found that a high-fat diet caused a more significant cardiac redox balance disruption than the OVX+SD group. Both RSW and E2 increased cardiac TAC activity in both diet groups, and no difference was observed between the effects of these two treatments. Furthermore, RSW enhanced the effects of E2 on increasing TAC cardiac levels in the SD and HFD groups. In addition, similar to the TAC cardiac levels, both treatments modulated the MDA cardiac level in the SD and HFD groups, and RSW was a suitable substitute for estrogen. In another part of the present study, we observed that the TAC/MDA ratio increased in the combined group, indicating that RSW enhanced the estrogenic effects. Therefore, RSW can be considered a good substitute for estrogen in modulating cardiac antioxidant factors in ovariectomized animals, similar to what was observed in the lipid profile.

Consistent with our study, evidence indicates that obesity increases cardiac levels of reactive oxygen species (ROS) and reduces antioxidant defenses (52). On the other hand, the decrease in estrogen levels due to ovariectomy can lead to dysregulation of antioxidant factors through increased lipid peroxidation and reduced antioxidant enzyme activity (53). Swimming has also been shown to effectively reduce oxidative stress-induced damage in the hearts of diabetic rats by decreasing cardiac MDA levels (54). Also, swimming can reduce cardiac oxidative stress through mechanisms such as decreasing PDE5 levels (55), increasing SOD activity (55), and signaling cGMP kinase (55).

Consistent with the current study’s findings, E2 treatment has been found to decrease cardiac oxidative stress in ovariectomized rats fed with HFD (56). A study indicated that, under menopausal conditions (low estrogen levels), there would be an increase in cardiac pro-oxidant factors (57). The reduction in estrogen levels following menopause and obesity can lead to elevated levels of MDA and TAC, both of which can be attenuated by E2 treatment (58, 59). The reduction in mitochondrial fission (58) and improved cardiac insulin resistance (56) are some of the mechanisms by which E2 alleviates cardiac oxidative stress.

Furthermore, in alignment with our results, it has been documented that eight weeks of swimming combined with estrogen treatment increases the expression of antioxidant enzymes and eNOS, which improve coronary artery disease (38). Almeida *et al*. have also demonstrated that regular aerobic exercise can serve as a substitute for E2 in reducing MI-induced SOD levels in the ovariectomized rats (39). However, in another study, E2 could not attenuate oxidative stress in the hearts of OVX animals and reverse the beneficial effects of aerobic exercise when combined with E2, possibly due to differences in type, duration, and dose of E2 therapy (60). Conclusion: Overall, an HFD in the postmenopausal animal model may increase body weight, BMI, visceral fat, and BG levels. However, these changes were ameliorated by RSW and E2 treatments, with the effects being more pronounced in the HFD group compared to the SD rats. On the other hand, the HFD increased TC, TG, and LDL levels and decreased HDL. However, these changes were equally prevented by RSW and E2, and swimming was only more effective than estrogen in increasing HDL levels in the SD group. We observed that RSW and E2 treatment corrected the reduced cardiac antioxidant activity induced by the HFD. Therefore, RSW improves metabolic cardiac risk factors in postmenopausal obesity, probably via the modulation of cardiac oxidative stress. Our study found that RSW could substitute for E2 in ovariectomized OVX animals. Future studies should investigate the mechanisms through which RSW might be a substitute for E2.

## Declaration

We have not used any AI tools or technologies to prepare this manuscript.
